# Characterization of the complete chloroplast genome of *Allium przewalskianum* (Amaryllidaceae)

**DOI:** 10.1080/23802359.2019.1698983

**Published:** 2019-12-12

**Authors:** Hongxia Wang, Bao Dong, Wenqi Li, Jiuli Wang, Wenjie Chen

**Affiliations:** a Key Laboratory of Biotechnology and Analysis and Test in Qinghai-Tibet Plateau, College of Ecological Environment and Resources, Qinghai Nationalities University, Xining, China;; b Key Laboratory of Resource Chemistry and Eco-environmental Protection in Qinghai-Tibet Plateau, Qinghai Nationalities University, State Ethnic Affairs Commission, Xining, China;; c Qinghai Provincial Key Laboratory of Crop Molecular Breeding, Key Laboratory of Adaptation and Evolution of Plateau Biota, Northwest Institute of Plateau Biology, Chinese Academy of Sciences, Xining, China

**Keywords:** *Allium przewalskianum*, chloroplast genome, phylogenetic tree

## Abstract

*Allium przewalskianum* (Amaryllidaceae) is an important vegetable and/or condiment for Tibetans, Indians, and Nepalese in the highlands of the Himalayas. Here, we reported the complete chloroplast genome of *A. przewalskianum* using the next-generation sequencing method. The size of the chloroplast genome is 153,509 bp in length, including a large single copy region (LSC) of 82,302 bp, a small single copy region (SSC) of 17,717 bp, and a pair of inverted repeat (IR) regions with 26,745 bp. The *A. przewalskianum* chloroplast genome encodes 114 genes, including 68 protein-coding genes, 38 tRNA genes, and eight rRNA genes. Phylogenetic tree analysis suggested that *A. przewalskianum* was closely related to *A.ampeloprasum* and *A.sativum.*

*Allium przewalskianum* (Amaryllidaceae) is a perennial herb widely distributed in the Qinghai-Tibet Plateau and adjacent areas, occurring at elevations between 1800 and 4500 m (Liang et al. 2015). It is an important vegetable and/or condiment for Tibetans, Indians, and Nepalese in the highlands of the Himalayas (Yao et al. 2011). This species is morphologically and genetically distinct from all the closely related species within the section *Rhizirideum* of the subgenus *Rhizirideum* (Zhou et al. 2006). In this study, we assembled the complete cp genome of *A. przewalskianum* (Genbank accession number: MN 519210) to provide genomic and genetic sources for further research.

The fresh leaves of *A. przewalskianum* were collected from Ping’an (101.91 E, 36.53 N), Qinghai Province, China. The genomic DNA was extracted following the modified CTAB from the leaf tissues (Doyle and Doyle 1987). The voucher specimen was deposited in Herbarium of the Northwest Institute of Plateau Biology (HNWP, WangHX2019002), Northwest Institute of Plateau Biology, Chinese Academy of Sciences. Genome sequencing was achieved on the Illumina HiSeq Platform (Illumina, San Diego, CA) at Genepioneer Biotechnologies Inc., Nanjing, China, and 6.9 GB of sequence data was generated. The low-quality reads and adapters were removed and high-quality reads were assembled via SPAdes (Bankevich et al. 2012). The assembled genome was annotated using CpGAVAS (Liu et al. 2012).

The chloroplast genome of *A. przewalskianum* was 153,509 bp in length, containing a large single copy region (LSC) of 82,302 bp, a small single copy region (SSC) of 17,717 bp, and a pair of inverted repeat (IR) regions of 26,745 bp. The circular genome contained 114 genes, including 68 protein-coding genes, 38 tRNA, and eight rRNA genes. The overall GC-content of the chloroplast genome was 35.37%, with the LSC, SSC, and IR regions being 34.73%, 29.60%, and 42.52%, respectively.

Phylogenetic analysis suggested that *A. przewalskianum* is closely clustered with *A. ampeloprasum* and *A. sativum* (Figure 1), which was generated based on the complete cp genome of *A. przewalskianum* and other species of the family Amaryllidaceae. Alignment was conducted using MAFFT (Katoh and Standley 2013). The phylogenetic tree was built using MEGA7 (Kumar et al. 2016) with bootstrap set to 10,000. This study could lay a foundation for population genomic studies, phylogenetic analyses, and genetic engineering studies of *A. przewalskianum* in the future.

**Figure 1.**
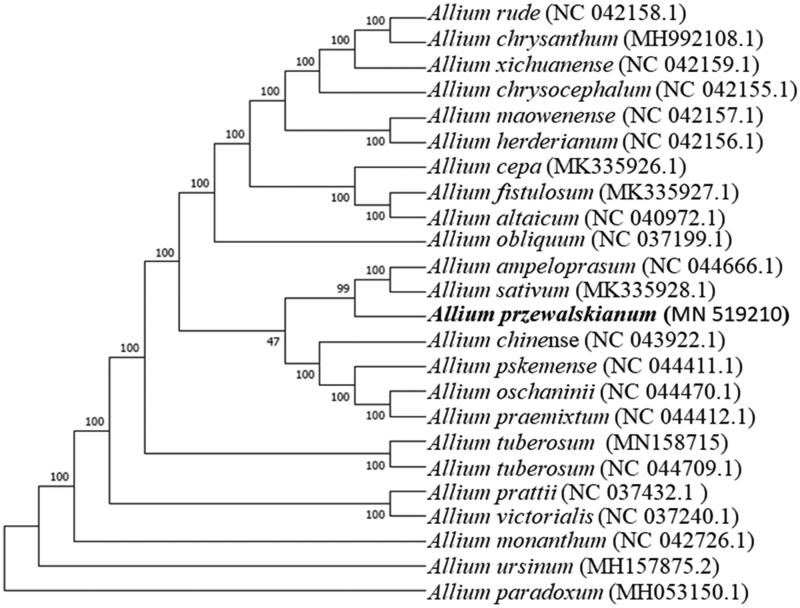
The ML tree based on 24 chloroplast genomes.

## References

[CIT0001] Bankevich A, Nurk S, Antipov D, Gurevich AA, Dvorkin M, Kulikov AS, Lesin VM, Nikolenko SI, Pham S, Prjibelski AD, et al. 2012. SPAdes: a new genome assembly algorithm and its applications to single-cell sequencing. J Comput Biol. 19(5):455–477. 22506599 10.1089/cmb.2012.0021 PMC3342519

[CIT0002] Doyle JJ, Doyle JL. 1987. A rapid DNA isolation procedure from small quantities of fresh leaf tissues. Phytochem Bull. 19:11–15.

[CIT0003] Katoh K, Standley DM. 2013. MAFFT multiple sequence alignment software version 7: improvements in performance and usability. Mol Biol Evol. 30(4):772–780. 23329690 10.1093/molbev/mst010 PMC3603318

[CIT0004] Kumar S, Stecher G, Tamura K. 2016. MEGA7: molecular evolutionary genetics analysis version 7.0 for bigger datasets. Mol Biol Evol. 33(7):1870–1874. 27004904 10.1093/molbev/msw054 PMC8210823

[CIT0005] Liang QL, Hu XX, Wu GL, Liu JQ. 2015. Cryptic and repeated “allopolyploid” speciation within *Allium przewalskianum* Regel. (Alliaceae) from the Qinghai-Tibet Plateau. Org Divers Evol. 15(2):265–276.

[CIT0006] Liu C, Shi L, Zhu Y, Chen H, Zhang J, Lin X, Guan X. 2012. CpGAVAS, an integrated web server for the annotation, visualization, analysis, and GenBank submission of completely sequenced chloroplast genome sequences. BMC Genomics. 13(1):715. 23256920 10.1186/1471-2164-13-715 PMC3543216

[CIT0007] Yao BQ, Deng JM, Liu JQ. 2011. Variations between diploids and tetraploids of *Allium przewalskianum*, an important vegetable and/or condiment in the himalayas. Chem Biodivers. 8(4):686–691. 21480514 10.1002/cbdv.201000305

[CIT0008] Zhou SD, He XJ, Ge S. 2006. trn-K-genebasedmolecular phylogeny of *Allium* plants (Lilaceae s.l.). Acta Bot Boreal-Occidenta Sin. 26:906–914.

